# Non-Adherence to WHO Recommendations Regarding Infant Feeding Practices Results in Dilemma of Malnourishment: A Community-Based Prospective Cohort Study Conducted in Karachi, Pakistan

**DOI:** 10.7759/cureus.8507

**Published:** 2020-06-08

**Authors:** Muhammad Osama, Anosh Aslam Khan, Sohaib Hasan Syed, Osama Mohiuddin, Ammar Hassan, Syeda Ramsha Zaidi, Neelofar Sami

**Affiliations:** 1 General Surgery, Dow University of Health Sciences, Karachi, PAK; 2 Internal Medicine, Dow University of Health Sciences, Karachi, PAK; 3 Internal Medicine, St. Mary Mercy Hospital, Livonia, USA; 4 Internal Medicine, Dow Medical College, Dow University of Health Sciences, Karachi, PAK; 5 Community Medicine, Aga Khan University Hospital, Karachi, PAK

**Keywords:** breastfeeding, infant mortality, infant feeding, malnourishment, nutrition, who guidelines

## Abstract

Background

The prevalence of chronic malnutrition and its associated morbid outcomes has been a significant cause of health loss globally, affecting millions of children hampering their mental, physical, social, and immune system development. World Health Organization's (WHO) recommendations presenting infant feeding guidelines have largely controlled this burden. However, developing countries including Pakistan have failed to promote these guidelines and still succumb to a huge burden of morbidity and mortality secondary to malnourishment among infants.

Methodology

Our study is a prospective cohort including 300 infants without predisposing congenital anomaly, followed from 6 months to 18 months of age. The primary outcome involved was classifying patients as malnourished based on anthropometric measurements, assessing the prevalence of co-morbidities and comparison of results in compliance with WHO guidelines.

Results

A total of 276 infants were included and the rest were lost to follow-up. Stratification on socioeconomic status was done; 53% of infants were diagnosed as malnourished, either due to stunted growth, underweight, or both. The odds of development of malnourishment based on non-adherence to WHO guidelines on breastfeeding were 2.87 (p=0.001). The incidence of morbid complications was higher in the malnourished group, including gastrointestinal and respiratory tract infections.

Conclusion

The implementation of WHO recommendations on infant feeding techniques can prove to be a pivotal instrument to control the soaring index of morbidities and mortalities associated with malnourishment. A strong focus on parental education and awareness among masses is required for its promulgation and controlling the infant health burden linked to this preventable condition.

## Introduction

Childhood malnutrition is defined as deficiency of nutrients and energy in diet, leading up to the point that body measurements including weight-for-age (underweight), height-for-age (stunting), and weight-for-height (wasting) fall at least two standard deviations below the mean. Childhood malnutrition, particularly during the first five years of life, is currently the single major contributor to the global burden of undernourishment [1,2]. Malnutrition among children is responsible for many serious consequences including growth failure in terms of stunted growth, delayed motor, cognitive and behavioral development, diminished immunity, and increased morbidity and mortality [3]. This is attributed to the fact that daily nutrients, such as carbohydrates, proteins, fat, and other micronutrients required to maintain the normal physiological functions such as visual acuity, immunity, etc, become scarce that predisposes the body to morbidities and their associated complications. The commonly prevalent morbidities associated with malnutrition are recurrent infections leading to debilitating heath consequences including diarrhea, pneumonia, and respiratory tract infections (RTIs) [2-4].

In year 2000, the fraction of total global health loss attributable to under-nutrition was 9.5%, while it was 14.9% in high-mortality developing regions [5]. By 2015, over three-fourths of the world’s malnourished children were living in the Southeast Asia region of the world, among which half of them died due to complications of malnutrition. About 53% of these deaths were attributable to underweight, more than 45% related to measles, and 63% of deaths were due to diarrhea [6,7]. This became a concern for public health practitioners, which needed to be addressed as a primary priority. Henceforth, the foremost target of the Millennium Development Goal was to ‘eradicate extreme poverty and hunger’ that will help in solving the devastating situation of childhood undernutrition [8].

The World Health Organization (WHO) Global Database on Child Growth and Malnutrition worked to collect, standardize, and disseminate anthropometric data on children based on a standard format. This helped in monitoring trends towards Millennium Development Goal achievement [7,9]. Henceforth, WHO has devised comprehensive recommendations to improve the nutritional status of children, which is predominantly based on height for age and weight for age [10]. Short height for age is referred to as stunting while the reduced weight for age is referred to as underweight. Both of these indicators reflect poor nutritional status. According to the guidelines, children between the age of 6 and 12 months should have a complementary diet in the form of thickened gruel at least three times a day along with breastfeeding till two years of age or at least one year of life [10,11].

Pakistan being one of the developing countries of Southeast Asia is also tremendously affected by the prevalence of childhood malnourishment. Over 11 million under-five children have been reported to have stunted growth by year 2011 [12]. Herein, we evaluate the impacts of following WHO’s feeding recommendation on growth indicators of children and the degree of reduction in malnutrition-associated complications such as diarrhea, pneumonia, and RTIs.

## Materials and methods

A prospective cohort study was conducted to observe the impacts of following WHO guidelines for exclusive breastfeeding and weaning practices on the nutritional status of children. The study design was approved without any objections by the institutional review board of Aga Khan University, Pakistan.

Inclusion and exclusion criteria

Three hundred predominantly breastfed infants aged six months were enrolled for the study from two squatter settlements of Karachi, Pakistan. Eligible infants were identified through data available from Lady Health Workers of the National Program for Family Planning and Primary Health Care, which was initiated by the Government of Pakistan in 1994. Based on the inclusion criterion, informed consent was taken from the mothers/caregiver of the enrolled infants. All the enrolled infants were given a project identification number (ID). Infants diagnosed with any congenital anomaly confirmed by reviewing birth certificates and relevant medical records before enrollment or for whom consent was not provided were excluded from the study.

Data points, stratification, and outcomes

Field staff hired for the study were given formal training on research ethics, obtaining informed consent, recruitment and enrollment of mothers and infants, use of educational and informational materials regarding common peadiatric morbidities with their preventive and management regimen corresponding to WHO's guidelines, administration of data forms, and taking anthropometric measurements at the time of enrollment, which included naked weight, recumbent length, and head circumference. The WHO recommendations were also translated into the native language of 'Urdu' for proper understanding of the subjects, while questionnaires for collecting data were designed separately. Educational materials and questionnaires were pretested in a field site that was socioeconomically similar to the field sites of the corresponding study.

Initially, a baseline survey was conducted by the field workers with the mothers/caregivers of the enrolled infants to obtain information regarding the name, participant’s ID number, infant’s date of birth, birth history, and birth weight (if available), breastfeeding, weaning practices, and socioeconomic indicators. Thereafter, they were responsible for visiting the household of infants once weekly to conduct counseling sessions regarding WHO nutritional recommendations, all the while encouraging mothers/caregivers to incorporate them in the regular dietary routine of the children. Additional guidance about hygiene, immunization, appropriate management, and timely referrals in case of any morbidity faced by the infants was also provided through these community-based workers. Information regarding breastfeeding practices, weaning practices, numbers/types of complementary feeds, consistency of food, and the occurrence of any morbidities like diarrhea, pneumonia, RTIs, and others was obtained on weekly basis during each visit.

At the end of one year of enrollment, when the infants reached the age of 18 months, anthropometric measurements were taken again by the trained field staff to notice underweight and stunting among children.

Statistical analysis

For statistical analysis, Z-scores for weight and height (weight-for-age, WAZ; height-for-age, HAZ; and weight-for-height, WHZ) were calculated at 6 and 18 months of follow-up with EPI Info^TM^ 6 (Centers for Disease Control and Prevention, Atlanta, GA) using the WHO Child Growth Standards and the National Center for Health Statistics [13]. The enrolled children were categorized into two groups, normal and malnourished, based on Z-scores. Malnourishment involves both stunted growth and underweight. Underweight was defined as having a Z-score of <-2 for WHZ, while stunted growth was defined as a Z-score of <-2 for HAZ.

A multivariate logistic regression analysis was applied to calculate the difference between the two groups and the prevalence of co-morbidities. We calculated the sample size to have a 90% probability at a 5% signiﬁcance level if the true difference between the groups was 0.02 units in linear growth velocity, based on an assumption that the standard deviation in the response variable is 0.05.

The data were analysed using SPSS for Windows, Version 24.0 (IBM Corp., Armonk, NY). Descriptive statistics and testing of hypothesis were applied for the statistical methodology. The univariate and multiple logistic regression methods were used to examine the association between different variables. A p-value of <0.05 was used to establish statistical significance.

## Results

Among the 300 recruited infants, 276 (92%) completed the study and could be followed up by the end of the study period. Twenty-four infants (8%) dropped out during the intervention study for the following reasons: family migration (12 cases), mother’s withdrawal from the study (eight cases), and death of the infants (four cases). Subjects were comparable in most of the parental and socioeconomic background.

Anthropometric measures were normalized, and based on a cut-off of two Z-scores below mean, infants were categorized as either malnourished, which included stunted and underweight infants, or as normal. Both of these groups were then standardized for comparison of variables: 147 infants (53.3%) were found to be malnourished and the remaining 129 infants (46.7%) were normal. Table 1 gives details about gender differences among normal and malnourished groups.

**Table 1:** Frequency of malnourished conditions categorized by gender Total number of infants, N=276

Variables	Males		Females	
	N	%	N	%
Normal	69	53.5	60	46.5
Stunted	42	28.6	51	34.7
Underweight	19	12.9	9	6.1
Both stunted and underweight	18	12.2	8	5.4

Among the two groups, 48.3% of the malnourished children were strictly breastfed until 18 months of age, and rest did not adhere to guidelines, comparable to 72.9% of the healthy infants (p=0.001). The analysis showed the odds of malnourishment to be 2.87 [1.73-4.76] as a result of non-adherence to WHO guidelines. Furthermore, the caregivers of only 16.3% of the undernourished children followed the instructions for administering complementary solid food at least three times a day. In contrast, 67.0% of normal children were fed with solid food at least three times a day. More details about dietary practices are described in Table 2.

**Table 2:** Dietary practices of malnourished and normal infants

Characteristics	Malnourished infants (N=147)		Normal infants (N=129)		p-value	Odds ratio
	N	%	N	%		
Breastfeeding for 18 months	71	48.3	94	72.9	0.001	2.87 [1.73-4.76]
Thick gruel	24	16.3	66	51.2	0.03	2.37 [2.07-4.37]
Variation in food content	50	34.0	98	76.0	0.04	1.40 [1.12-3.76]
Administered solid food 3 times or more	24	16.3	88	67.0	<0.001	2.40 [2.08-4.52]

Table 3 describes the characteristic differences in morbidities among the two groups in terms of frequency and incidence. The malnourished children were more likely to have diarrhea than normal ones, with the incidence being 67.9 per 1000 compared to the incidence of 58.2 per 1000 among normal infants (p=0.02). Upper respiratory tract infections were also observed to be significantly more prevalent in the malnourished group (p=0.04).

**Table 3:** Incidences of individual morbidities among normal and undernourished children *Incidence rate = (Total number of new cases during a given time period/Total population at risk during the same time period)*1000

Morbidity and incidences	Normal children (N=129)	Undernourished children (N=147)	p-value
Total episodes of diarrhea	425	456	
Incidence*	58.2	67.9	0.02
Total episodes of pneumonia	83	74	
Incidence*	10.9	11.0	0.46
Total episodes of respiratory tract infections	215	250	
Incidence*	30.7	37.3	0.04

## Discussion

One of the greatest dilemmas of lower and middle-income countries is the predominant prevalence of maternal and childhood malnutrition that has led to a grave impact on the survival rate, public health index, incidence of acute and chronic diseases, and economic productivity of a single individual as well as societies at large [14]. Despite the fact that developed regions of the world have made accelerating progress in controlling the under-five mortality rate, by 2018, Sub-Saharan Africa and Central and Southern Asia were reported to suffer from over five million deaths of children under five years of age. Around 50% of all these deaths occurred in just five countries, namely, India, Pakistan, Nigeria, Ethiopia, and the Democratic Republic of Congo. The most noteworthy contributor to under-five mortality was prevalent malnourishment, roughly accounting for 45% of deaths [15].

Pakistan is one of the main South Asian countries that has suffered persistently from a heavy burden of malnourishment in children. Our study is conducted in Karachi, the most densely populated and largest cosmopolitan city of Pakistan. More than 50% of the observed subjects were experiencing the dilemma of malnutrition. This parallels with other studies conducted in different regions of Asia. A study conducted in Nepal reported that approximately 70% of the world’s malnourished children live in Asia [4]. Growth failure is defined as underweight, stunting, and wasting [16]. Throughout our study, we have scrutinized two major complications of malnourishment, i.e. stunting and underweight.

About one-third of the children were observed to be underweight or stunted by the end of our study period. Özaltin et al. presented a thorough data analyzing growth failure in children from 54 various low- and middle-income countries [17]. The research showed that over 38% of the children were suffering from stunting, which is similar to the findings of our study. However, more than one-fifth of the children were underweight, which is comparatively less than our observations. An analysis for the Lancet Nutrition Series based on the population-based surveys from 79 countries demonstrated that stunting prevailed 2.4 times more in the poorest quintile of households as compared to richest households. This is compounded by the fact that the highest prevalence of stunting was observed in East Africa (42%) and South-Central Asia (36%) [16]. Bhutta et al. had estimated an increase in the number of stunted under-five children in Pakistan from 6.35 million to over 11 million from 1985 to 2011 [12]. However, China, a northern Pakistan’s neighbor, demonstrated a marked decline of 70% in stunting of children from 1975 to 2010. Studies have shown a positive correlation between the improvement of stunted growth with gross domestic product (GDP) per capita and urbanization [18]. Meanwhile, Pakistan is still struggling toward financial and economic prosperity.

Concerning the children being underweight, the United Nations has an estimated global prevalence of 16%, among which the highest number of children were again found in several countries of South-Central Asia and Western Africa by 30% and 22%, respectively [14]. Like Pakistan, several countries have been included under the category of very high prevalence level of underweight (more than 30%) in under-five children. These countries include Bangladesh, Bhutan, Yemen, Ethiopia, India, Indonesia, Maldives, Madagascar, Myanmar, Nepal, Nigeria, and Sudan, all of which belong to the developing or underdeveloped regions of the world [9].

Keeping in view such a high prevalence of malnutrition among children and the resultant growth failure, the WHO had come up with recommendations regarding dietary routine during one to five years of life, to prevent the dilemma of malnourishment among children [11]. The results of our study illustrate that these recommendations of the WHO were not followed properly in the dietary habits of undernourished children. In contrast, normal children were observed to be following the WHO guidelines appropriately. We have deeply studied the four major guidelines of WHO in our survey.

Concerning the recommendation about breastfeeding until two years of age, our results showed a positive association between breastfeeding until two years of age and weight gain along with linear growth among infants. Parallel trends have been evident in other studies conducted in Kenya and Philippines showing a significant relationship of lack of exclusive breastfeeding after six months of age with underweight and stunting among children [19,20]. A study conducted in England revealed that the children breastfed until 18 months of age showed better physical and cognitive development as compared to those whose breastfeeding was discontinued before 18 months of age [21]. Hence, these findings prove the importance of breastfeeding until 18 months for enhanced physical and cognitive development of a child.

Regarding the guideline of introducing a variety of nutritious food to children who have started a complementary diet in the form of thickened gruels, a positive correlation between growth of infants and diversity of food is observed in our study analysis. This is quite similar to a study conducted in Kenya illustrating that providing a variety of food to growing infants prevents undernourishment [19]. A survey from 11 different developing regions involving children 6-23 months of age demonstrates that dietary diversity is significantly associated with the growth of children, lack of which can create barriers in the normal development [22]. Several studies, some of which were conducted in Sub-Saharan Africa, showed that a better food diversity index was associated with improved nutritional status in children [22-24]. In most Asian countries, the contents of the gruels mostly include sweet potato, cereals, maize, barley, oat, etc. Studies held in Congo and Bangladesh also point out that the introduction of thickened gruel formed from a different variety of native food makes a healthier contribution to the child’s normal development [25-27].

Our study also shows that the daily administration of solid food three times or more per day, as per the guidelines of WHO, makes a noteworthy contribution to the physical development of children. Analysis of our study runs parallel to research conducted in Bangladesh that provides strong evidence for the positive effects of practicing WHO infant feeding recommendations on the growth of infants and young children and hence, signifies the importance of this guideline [25].

Last but not the least, malnutrition is associated with several life-threatening morbidities that eventually set forth a vicious cycle of severe malnourishment. The results of the present study demonstrate that a significantly higher risk of morbidities, including diarrhea, pneumonia, and RTIs, is associated with undernourished children. Rodriguez et al. also pointed out that a greater proportion of gastrointestinal infections, respiratory tracts infections, diarrhea, and immune suppression are few of the many grave outcomes of chronic malnourishment. These complications often contribute in further reduction of food intake [2].

All in all, malnutrition impacts several aspects of a child’s growth including health, cognition motor, and social development. It is one of the most fundamental causes of morbidities and has attributed to nearly 50% of mortality among affected children. In a developing country like Pakistan, interconnecting themes of early marriages, large family unit, high fertility rate, reduced birth spacing, low income, lack of exclusive breastfeeding, and improper complementary diets have set the foundation for chronic malnourishment in young children. In the case of developing and poor-income regions, socioeconomic status and demographic factors also play a considerable role in the nutritional status of a child apart from the proper adherence to infant feeding guidelines of WHO [28-30]. A similar picture is portrayed in our study as it is conducted in squatter settlements of Karachi where most infants belong to poor and middle-class families with weak economic background and substandard hygienic conditions, thus potentiating the devastating results of malnourishment.

## Conclusions

The findings of our study provide strong evidence to incorporate the current infant feeding recommendations of the WHO in the dietary habits of a child for healthier physical and cognitive development during infancy and early childhood. Since malnutrition is associated with high rates of morbidities and mortalities among infants, the implementation of WHO recommendations can prove to be a pivotal instrument in controlling the soaring index of morbidities and mortalities. The promotion of feeding recommendations at the grass roots is required especially in communities of low socioeconomic status. Efforts should be made at community levels to educate the caregivers/mothers to adhere to these guidelines and spread awareness about grave outcomes of non-adherence. National and local authorities should consider taking essential steps to ensure adequate resources and food for infant feeding and healthcare. Proper comprehension and adherence to these guidelines by the mothers and caregivers will prove an effective tool to address the burden of undernutrition among infants in Pakistan and elsewhere in the world.
